# SHM System for Composite Material Based on Lamb Waves and Using Machine Learning on Hardware

**DOI:** 10.3390/s24237817

**Published:** 2024-12-06

**Authors:** Gracieth Cavalcanti Batista, Carl-Mikael Zetterling, Johnny Öberg, Osamu Saotome

**Affiliations:** 1 KTH Royal Institute of Technology, School of Electrical Engineering and Computer Science, 164 40 Kista, Swedenjohnnyob@kth.se (J.Ö.); 2 ITA Technological Institute of Aeronautics, Electronic and Computer Engineering, São José dos Campos 12228-900, SP, Brazil; osaotome@ita.br

**Keywords:** structural health monitoring, machine learning, composite material, outlier solution, hardware implementation

## Abstract

There is extensive use of nondestructive test (NDT) inspections on aircraft, and many techniques nowadays exist to inspect failures and cracks in their structures. Moreover, NDT inspections are part of a more general structural health monitoring (SHM) system, where cutting-edge technologies are needed as powerful resources to achieve high performance. The high-performance aspects of SHM systems are response time, power consumption, and usability, which are difficult to achieve because of the system’s complexity. Then, it is even more challenging to develop a real-time low-power SHM system. Today, the ideal process is for structural health information extraction to be completed on the flight; however, the defects and damage are quantitatively made offline and on the ground, and sometimes, the respective procedure test is applied later on the ground, after the flight. For this reason, the present paper introduces an FPGA-based intelligent SHM system that processes Lamb wave signals using piezoelectric sensors to detect, classify, and locate damage in composite structures. The system employs machine learning (ML), specifically support vector machines (SVM), to classify damage while addressing outlier challenges with the Mahalanobis distance during the classification phase. To process the complex Lamb wave signals, the system incorporates well-known signal processing (DSP) techniques, including power spectrum density (PSD), wavelet transform, and Principal Component Analysis (PCA), for noise reduction, feature extraction, and data compression. These techniques enable the system to handle material anisotropy and mitigate the effects of edge reflections and mode conversions. Damage is quantitatively evaluated with classification accuracies of 96.25% for internal defects and 97.5% for external defects, with localization achieved by associating receiver positions with damage occurrence. This robust system is validated through experiments and demonstrates its potential for real-time applications in aerospace composite structures, addressing challenges related to material complexity, outliers, and scalable hardware implementation for larger sensor networks.

## 1. Introduction

In aircraft design, it is essential to account for the diverse stresses that structural materials undergo, such as torsion, bending, tensile, shear, and compressive forces. For example, composite materials used in wings must endure substantial bending loads, especially within the spars, as the aircraft is in flight. This analysis is a foundational aspect of aircraft engineering, requiring meticulous and detailed evaluation [1].

Aircraft maintenance adds another layer of intricacy, as it often demands a considerable investment of time and resources, including the partial disassembly of major structural elements to perform manual inspections [2]. Nondestructive testing (NDT) methods have become indispensable tools in tackling these challenges [3,4]. NDT approaches offer cost-effective ways to conduct preventive, predictive, and corrective maintenance, as they allow for detecting small defects without impairing the component’s integrity [5]. These techniques are applied throughout the manufacturing process to ensure defect-free parts and are used during the aircraft’s operational life to detect cracks as fine as 1 mm. For instance, inspectors may focus on examining fastener holes at critical junctions between the wing and spar, which are susceptible to damage, while leaving other areas uninspected.

Fastener holes at junction areas in aerospace structures are highly susceptible to damage extension due to the stress concentrations they create and the fatigue effects from cyclic loading. These stress risers often lead to crack initiation and propagation, compromising structural integrity over time. Composite materials in aerospace commonly utilize adhesive bonding and mechanical fastening for joining. Adhesive bonding ensures uniform load distribution, avoids stress concentrations, and is sensitive to surface preparation and environmental factors. While reliable and easy to inspect, mechanical fastening introduces stress concentrations around drilled holes, increasing the risk of delamination and fatigue. Combining both techniques, hybrid joining methods are often employed to balance durability, performance, and ease of maintenance in critical composite joints [3,6]. Moreover, co-curing and co-bonding provide integrated solutions by bonding composite layers during curing and enhancing joint strength while reducing additional weight. Thermoplastic welding, including ultrasonic and induction welding, enables the efficient and lightweight fusion of thermoplastic composites without adhesives or fasteners. Z-pinning often enhances delamination resistance by reinforcing the through-thickness direction with inserted pins [6]. However, working at junction areas does not fall under the scope of the system described in the present paper.

Non-destructive testing (NDT) and structural health monitoring (SHM) serve distinct purposes in evaluating the integrity of composite materials. NDT refers to a collection of inspection techniques, such as ultrasonic testing, thermography, and neutron imaging, which detect defects or damages in composite structures without causing any harm to the material. These methods are typically employed during and after manufacturing, and even after mechanical testing, to ensure structural integrity. On the other hand, SHM focuses on the real-time or online monitoring of damage progression within a composite structure, particularly during operational or mechanical loading. Unlike NDT, SHM methods, such as acoustic emission testing and electrical impedance measurements, actively observe the development and propagation of damage in response to stress. For instance, acoustic emission techniques detect stress waves generated during crack formation, continuously monitoring damage events. However, they are not classified as NDT since they often operate in destructive testing scenarios [7,8,9]. Similarly, SHM, using impedance measurements, monitors damage during loading by analyzing changes in the electrical properties of nanocomposite adhesives [8]. Thus, while NDT emphasizes defect detection in intact structures, SHM targets the ongoing behavior and extension of damage under applied loads [9].

Modern SHM systems are increasingly designed to operate with minimal power, offering real-time data while the aircraft operates [5]. This presents a significant engineering challenge as researchers strive to create effective and efficient solutions. The trend toward online, energy-efficient SHM systems reflects ongoing progress in this field to enhance aircraft safety and structural reliability [10].

A range of current inspection methods are utilized for aircraft structures, including liquid penetrant inspection, magnetic particle testing, eddy current analysis, ultrasonic testing, radiographic (X-ray/gamma-ray) methods, visual/optical assessments, resonance testing, and infrared thermography [11,12]. Zahedi and Huang [13] proposed a technique to improve damage detection in SHM by employing piezoelectric wafer active sensors (PWAS). Their method involves converting electromechanical impedance (EMI) signatures from the frequency domain into the time domain via digital signal processing, while treating the EMI signature as a pulse-echo signal. This conversion separates the signal’s excitation, resonant, and echo phases, thus enabling the more precise localization and characterization of structural and bonding issues. The approach significantly enhances damage sensitivity, allowing for more apparent distinction among damage types.

Xu et al. [14] applied a particle swarm optimization–support vector machine (PSO-SVM) hybrid model to assess damage in polymer cables, with an accuracy of 77% in noisy environments. However, the hybrid approach lacks specialized outlier handling capabilities.

Moreover, it is common for systems based on sensor data acquisition to have outliers, which is challenging for the ML decision maker. In the case of SVMs, researchers have applied Mahalanobis distance to deal with many problems, mainly the outlier problem. For example, Goyal et al. [15] proposed a low-cost, non-contact vibration sensor for detecting faults in bearings. Combined with SVM for classification, this sensor offers an effective alternative to traditional accelerometers for bearing fault diagnosis in rotating machinery. The Mahalanobis distance in this paper serves as a key feature selection technique. It allows the authors to identify the most relevant features from the extracted vibration data for fault classification, effectively reducing dimensionality and improving the robustness of the model. In the outlier topic, Dashdondov and Kim [16] used the Mahalanobis distance to remove multivariate outliers from health datasets, enhancing the accuracy of hypertension prediction models. This method enabled the authors to refine the dataset by excluding extreme outlier values, thus creating a cleaner and more representative training set for machine learning models.

In the present paper, an SHM system has been designed to detect and differentiate between internal and external defects within an aircraft structure. A machine learning approach, the support vector machine, facilitates the characterization of these defects, where the inference stage has been specifically optimized for identifying outliers. A piezoelectric (PZT) sensor transmits Lamb waves to the composite material, and four PZT receivers acquire the vibration reflection that navigates through the plate. A data acquisition sub-system generates, receives, and controls these signals, and the digital signal processing (DSP) step extracts meaningful information from the received signals. Then, we have an outlier detection phase that differentiates the data points with the highest standard deviation from the dataset mean. Finally, there are the training and inference phases of the support vector machine (SVM) algorithm, where we have an optimized implementation in hardware for the outlier cases so they are not ignored or misclassified.

The contributions of the proposed system can be evaluated in three aspects:

- Data Handling and Acquisition Methods: It utilizes Lamb waves generated by piezoelectric sensors to assess internal and external defects in composite plates, specifically addressing the SHM needs for real-time monitoring using FPGA hardware. The setup involves precision in frequency selection and positioning to optimize detection while minimizing interference. The system can cover an area of 60 cm × 60 cm in only one scanning, which can take up to 5 s (considering the sensing data acquisition followed by the damage information processing) to reach its first response.
- SVM Implementation and Outlier Management: It implements an optimized SVM using Mahalanobis distance for enhanced outlier handling, as the data variability in composite materials can be high. This enhancement aids in minimizing misclassifications and improving detection accuracy.
- Result Metrics and Performance Evaluation: It achieved high accuracy in defect detection, with success rates of 96.25% for internal defects and 97.5% for external defects, demonstrating the efficacy of Mahalanobis-enhanced SVM in real-time SHM applications. The FPGA hardware implementation also provides a practical balance between detection performance and latency.

## 2. Related Works

Chen et al. [17] developed a Lamb wave-based SHM system that accurately localized multiple damages across complex composite plate configurations with a 95% accuracy rate. However, their system is not designed for real-time applications and relies on offline data processing, unlike the proposed FPGA-optimized SVM approach, which provides immediate damage detection and localization, making it more suitable for dynamic SHM environments. Gao’s method [18] involves complex neural network structures and requires more computational resources, whereas the proposed Mahalanobis-enhanced SVM model is computationally efficient on FPGA hardware, making it ideal for real-time SHM without extensive processing overhead. Xu et al. [14] applied a PSO-SVM hybrid model to assess damage in polymer cables, with an accuracy of 77% in noisy environments. However, the hybrid approach lacks specialized outlier handling capabilities.

Liu et al. [19] proposed a study on how varying debonding lengths and directions impact the signal amplitude and phase in both simulation and experimental setups. The main results reveal that debonding affects the voltage distribution and signal amplitude, but the changes depend on the length and orientation of the debonding. For instance, signal amplitude does not consistently decrease with increasing debonding length, and debonding in different directions yields distinct effects. The authors suggest that future work should explore more comprehensive models that account for other environmental and mechanical factors to improve SHM reliability.

Tang et al. [20] introduced a machine learning approach using sparse sensor arrays combined with multi-domain feature extraction and SVMs for the accurate localization and classification of damage. Their study highlights the importance of Principal Component Analysis (PCA) for optimizing detection paths and ensuring data fusion. Similarly, Azuara et al. [21] employed wavelet transform and Convolutional Neural Networks (CNNs) to analyze Lamb wave signals for damage localization in composite plates. Their method achieved high precision with deviations below 15 mm, emphasizing the role of wavelet-based preprocessing and advanced imaging techniques in enhancing SHM.

Dabetwar et al. [22] leveraged cross-correlation and statistical feature extraction to enhance the performance of ML algorithms in classifying fatigue damage in composites. Rahbari et al. [23] proposed an unsupervised clustering framework for Lamb wave signals using dimensionality reduction techniques like t-SNE and UMAP combined with Deep Neural Networks (DNNs). These studies underscore the capability of ML to handle high-dimensional datasets and address the complexities of composite materials, offering robust solutions for identifying damage under varying conditions.

Fiborek et al. [24] developed a parallel spectral element method optimized for GPU computation to model guided wave propagation in composite structures, demonstrating its effectiveness in structural health monitoring (SHM) through model-assisted damage size estimation. Liu et al. [25] introduced a data-driven approach employing machine learning to characterize delamination damage in composite laminates, utilizing features such as Lamb wave propagation to predict damage extent accurately. Similarly, Liu et al. [26] proposed a prognostic framework using regression models like random forests to predict delamination growth trends, emphasizing the critical role of path length measurements for training data augmentation and improving model robustness. These studies underscore the integration of advanced computational techniques and machine learning for efficient and precise SHM in aerospace composites.

Applications of Lamb wave-based SHM have also extended to specific domains such as electrical insulation and anisotropic plates. Li et al. [27] focused on stator insulation damage in large generators, employing multi-feature fusion from time, frequency, and fractal domains integrated with SVM for precise damage identification. Moavenian et al. [28] also tackled acoustic source localization in anisotropic plates, demonstrating how low-sampling-rate sensors can effectively identify damage. These advancements illustrate the adaptability of Lamb wave techniques and their integration with ML across diverse engineering challenges.

Gao et al. [18] proposed a damage localization method for composite structures using Lamb wave signals and a Modular Artificial Neural Network (M-ANN). They aim to improve SHM by enabling accurate damage detection with fewer sensors. The main results demonstrate that the method achieves high-precision damage localization with a relative error of only 2.96% on average, outperforming other advanced techniques. The research highlights the potential for improved data acquisition methods and explores the model’s generalization to various damage sizes. Future work should focus on incorporating environmental factors and enhancing non-baseline damage diagnosis.

At the front-end part of the system, we have Tang, Mandal, and Özdemir’s proposal [29]: a highly integrated CMOS system-on-chip (SoC) designed for wireless ultrasonic power and data transfer, specifically for active SHM systems. This chip integrates an ultrasonic power management unit, a transceiver, and multiple DC-DC converters to facilitate energy-efficient, miniaturized wireless SHM nodes capable of transmitting data and detecting structural damage. The main results demonstrate high power efficiency (over 85% for the active rectifier and 70% for the inductive DC-DC converter) and successful wireless power/data transfer over long distances. However, future improvements are necessary to address environmental factors affecting power transmission and to further miniaturize the system by integrating additional components, such as an ADC and memory, onto the SoC. The future work will focus on enhancing power efficiency, reliability, and system integration to make it more applicable to diverse structural monitoring scenarios.

## 3. System Development: Data Acquisition

Each SHM system has unique specifications and requirements, often guided by the materials’ mechanical properties. These properties help determine the parameters to be analyzed. For this study, Finite Element Method (FEM) analysis was performed based on the material characteristics to identify an optimal vibration frequency for the plates that would not induce damage [30]. The analysis and frequency sweeping revealed that the ideal excitation frequency for the material’s first vibration mode is 15 kHz, and the asymmetric mode of Lamb waves is employed.

The system uses two specimens, specifically carbon fiber-reinforced plastic (CFRP) composite laminates, manufactured for KTH at Saab AB Aeronautics in Sweden. All laminates were fabricated using a 180°C cure epoxy prepreg (Hexply 6376) reinforced with unidirectional (UD) High Tenacity (HTS) carbon fiber, featuring a fiber volume content of approximately 57%. The prepreg had a cured ply thickness (CPT) of 0.129 mm. The laminates followed a quasi-isotropic (QI) lay-up configuration of [0, 90, 45, −45]_5_S, consisting of a total of 40 plies, resulting in a calculated overall thickness of 5.16 mm. The panel used in the experiments was trimmed by 10 mm along each edge using a diamond blade band saw to ensure precise and clean edges. This method was selected to minimize defects such as fiber pull-out, delamination, or micro-cracks that could arise during trimming, preserving the structural integrity of the composite edges. The use of a diamond blade is consistent with standard practices for preparing composite specimens, ensuring that edge quality does not compromise the propagation of Lamb waves or the accuracy of signal interpretation. This preparation step is critical for mitigating edge-related reflections and mode conversions that could affect the reliability of damage detection and classification in the SHM system.

**Figure 1:**
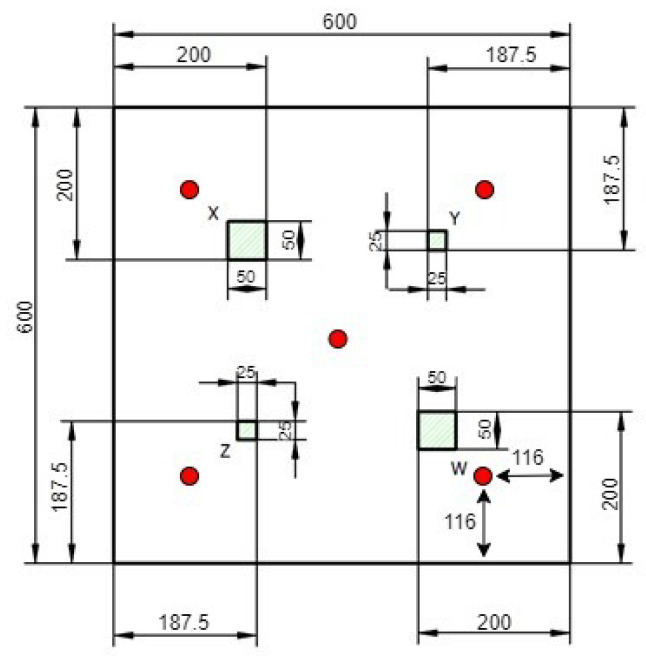
Specimen with internal defects.

**Figure 2:**
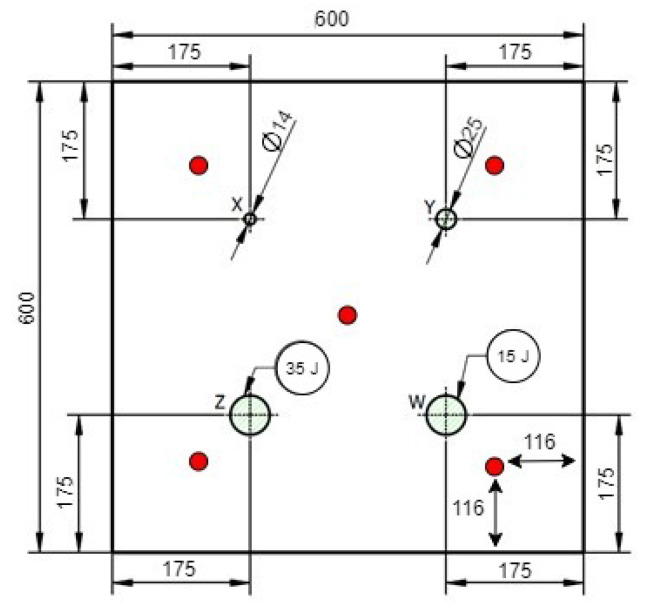
Specimen with defects on the surface.

Two laminates measuring 600 × 600 × 5 mm were produced: one laminate with embedded artificial defects (“Specimen Internal” shown in Figure 1) and another with visible surface defects (“Specimen External” as illustrated in Figure 2). Four defects were placed at the center of each quadrant in the damaged plates. The receiver sensors were located between the damages and the edges, with a distance of 116 mm from the edges (avoiding closeness to the edges because of the border effect and ensuring that their positions were well defined), as shown by the red dots at the middle of each quadrant in Figure 1 and Figure 2.

In the laminate with embedded defects of Figure 1, four defects, each including three layers of 2 mils thick FEP release film, were embedded at each quadrant of the specimen.

1. The defect X (50 × 50 mm) is created between layers 4 and 5 (from the bottom of the lay-up).
2. The defect Y (25 × 25 mm) is created between layers 32 and 33 (from the bottom of the lay-up).
3. The defect Z (25 × 25 mm) is created between layers 20 and 21 (from the bottom of the lay-up).
4. The defect W (50 × 50 mm) is created between layers 32 and 33 (from the bottom of the lay-up).

In the laminate with artificial defects, i.e., defects on the surface, one circle with Ø 25 mm and one circle with Ø 14 mm were cut out (before the cure of the laminate) in the top four plies at the locations drawn in Figure 2. One artificial impact damage with 15 J and one with 35 J were also made on the laminate.

All these defects were chosen based on the following reasons:

Artificial Defects (Circular Cutouts): Introducing circular cutouts in composite laminates is a common method to simulate damage scenarios such as delaminations or material removal. This approach is utilized to study the structural response and damage tolerance of composite materials. For instance, the ASTM D7136/D7136M standard [31] outlines procedures for impact testing on composite laminates, which often involve creating predefined defects to assess damage resistance.

Artificial Impact Damage (15 J and 35 J): Applying controlled impact energies to composite specimens is a standard practice to evaluate their impact resistance and damage tolerance. The ASTM D7136/D7136M standard [31] specifies methods for measuring the damage resistance of fiber-reinforced polymer matrix composites to drop-weight impact events, with energy levels like 15 J and 35 J being typical for such assessments.

Embedded Defects (Release Film Layers): Embedding release films within composite laminates to simulate delaminations or voids is a recognized technique in damage assessment studies. This method allows researchers to analyze the effects of internal defects on composites’ structural integrity. Studies such as “Defects Assessment of Composite Laminates from Scanning to Modeling” discuss the characterization of embedded defects and their impact on composite materials [32].

**Figure 3:**
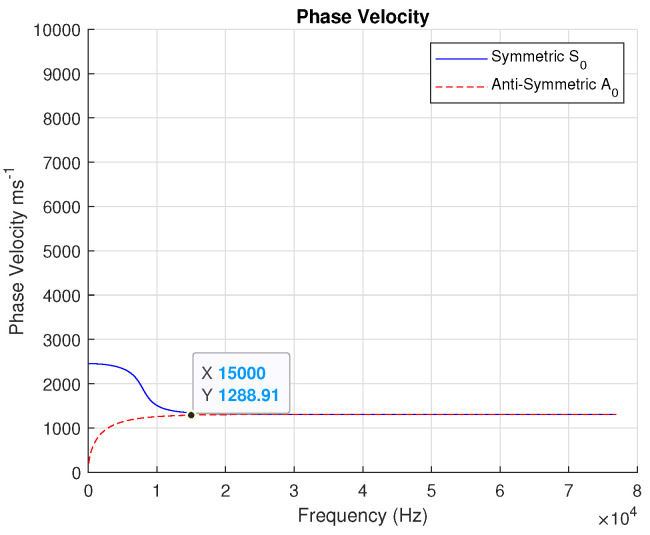
Lamb wave dispersion curve: phase velocity.

Then, analyzing the Lamb wave dispersion curves of the laminates, we have the phase and group velocities. From the phase velocity curve in Figure 3, it is possible to observe the following:

- S0 mode starts with a higher velocity at low frequencies, indicating that it is a more dominant mode for efficient energy transfer at long wavelengths.
- A0 mode starts with a lower velocity at low frequencies, characteristic of the flexural motion in Lamb waves.
- As the frequency increases, the phase velocity of both modes approaches a near-constant value, representing the higher frequency asymptotic behavior.

**Figure 4:**
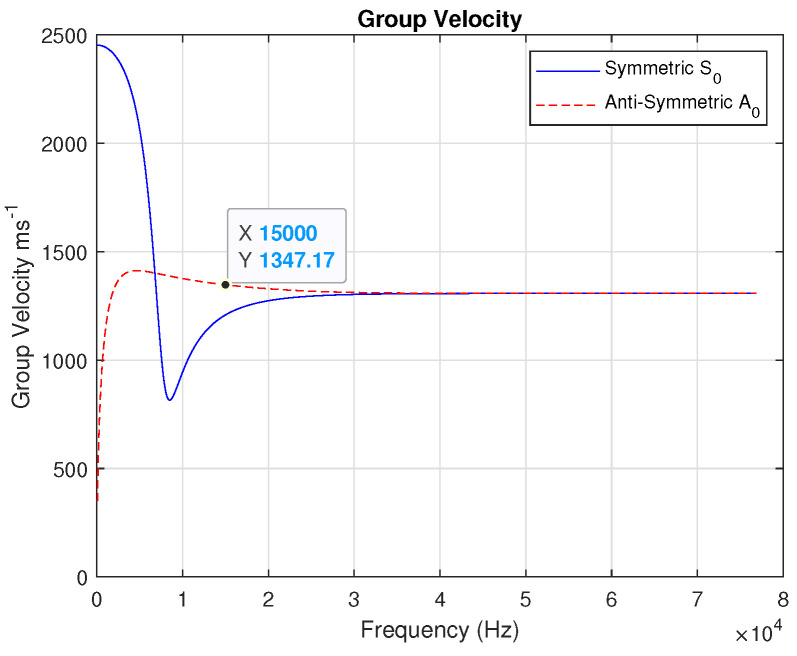
Lamb wave dispersion curve: group velocity.

From the group velocity curve in Figure 4, it is possible to observe the following:

- S0 mode shows an initial peak, followed by a dip, before stabilizing. This peak indicates a region where energy transfer is maximized.
- A0 mode displays a relatively flatter curve with slower stabilization than S0 mode.
- The stabilization of group velocity at higher frequencies suggests that energy propagation becomes more consistent as frequency increases.

The mode selection was based on the following observation:

- S0 mode is faster and typically preferred for applications requiring efficient energy transfer.
- A0 mode, due to its flexural nature, is often used for detecting surface defects or discontinuities.

Due to the SHM system’s proposed goal, we chose to work with the A0 mode because of the types of damage and their location (on the surface and between layers) in the specimens.

The excitation frequency at the transmitter of 15 kHz proves accurate because low frequencies are ideal for penetrating thick materials, which fits well in this case (see Figure 3 and Figure 4).

The selection of four receiver channels was based on empirical testing and was inspired by the configuration of sensor networks typically employed on flat structures, which is the target application for this system. The chosen layout places one transmitter at the center of four receivers, creating a localized sensing zone that allows the effective coverage of a larger wing area when extended across a network. This arrangement ensures that Lamb wave signals can be captured from multiple directions, improving damage localization accuracy while maintaining system simplicity.

**Figure 5:**
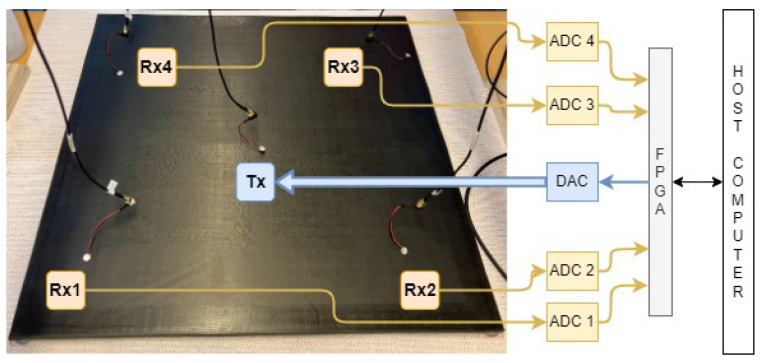
Overview of the data acquisition system with one transmitter and four receivers placed on the center and corners of the specimen, respectively.

The work described by Alan Alhallak [33] was implemented in this system, and it includes several key subsystems: a transmitter, four receivers, a DAC, four ADCs, an FPGA board, and a host computer, as illustrated in Figure 5. Mechanical Lamb waves are generated, captured, and sampled following the setup.

The transmitter subsystem generates Lamb waves and delivers them to the sensor via a Digital-to-Analog Converter (DAC). The DAC was used through a PmodDA3 board, which offers a 16-bit resolution, a 2.5 V reference voltage, and compatibility with the FPGA’s Pmod ports.

**Figure 6:**
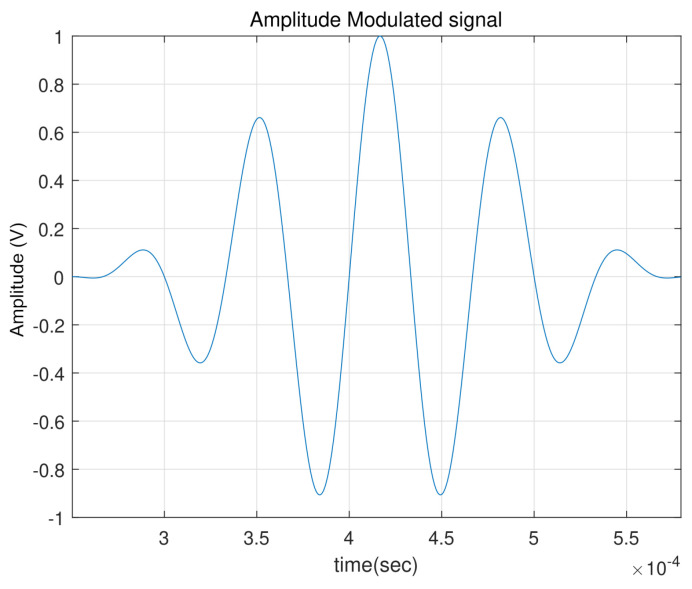
Actuation signal for the transmitted signal characterized by a 3.5−period and a 15 kHz sine wave, modulated by a 3 kHz Hanning window signal.

The transmitted signal, designed to vibrate the structure, was a 3.5-period sine wave at 15 kHz, modulated by a 3 kHz signal, as shown in Figure 6 [13,34].

The 15 kHz frequency was selected to remain sufficiently apart from the composite material’s natural frequency, thereby avoiding any potential damage and preventing interference with other aircraft systems.

The DAC, operating with a 2.5 V reference voltage, requires a power amplifier (PA) circuit to ensure that the transmitted signal effectively propagates through the material to reach the receivers. This PA circuit offers adjustable gain and offset for precise output tuning [35]. A PZT sensor, connected to the PA output and positioned at the center of the CFRP plate, generates the Lamb wave signal.

Each of the four PZT receivers is placed in a corner of the composite material. It detects vibrations and converts them into electrical signals. These signals are digitized by an Analog-to-Digital Converter (ADC). The chosen ADC board, incorporating an AD7091R chip on the EVAL-CN0350-PMDZ platform, meets the system’s requirements.

This ADC board features a maximum sampling rate of 1 MSPS, a 2.5 V reference voltage, and an offset voltage of 1.25 V, with a resolution of 12 bits and a 12-pin Pmod-compatible connector. A key feature is its conditioning stage, which is explicitly designed for piezoelectric sensors. It transforms the charge from the PZT sensor into a digital voltage, filters out noise, and amplifies the signal. The ADC board functions through three stages: filtering, amplification, and conversion via the ADC chip. To optimize signal quality, two resistor values were modified in the filtering and amplification stages to *R*3 =10 kΩ and R8=100 Ω (see CN0350 datasheet in [36]). The processed signals are subsequently stored on the FPGA.

The system uses a ZedBoard Zynq 7000 series FPGA device, which manages data transmission, reception, storage, processing, and transfer from the setup to a host computer. The board has two main components: the Programmable Logic (PL) and the Processing System (PS).

**Figure 7:**
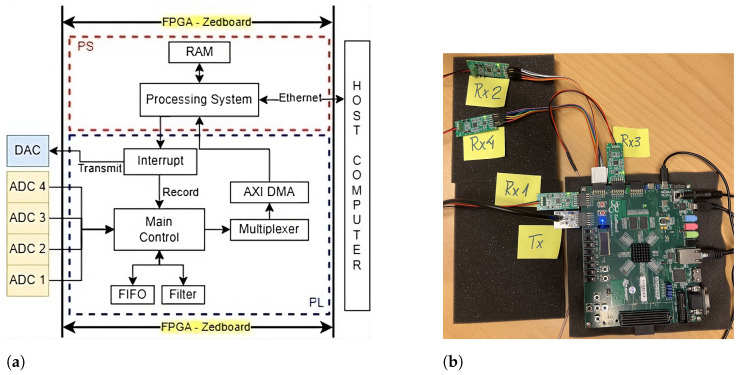
Data acquisition system. (**a**) Block diagram of the FPGA architecture design. (**b**) Devices used in the data acquisition system: four EVAL-CN0350-PMDZ boards (Rx1, Rx2, Rx3, and Rx4) and one PmodDA3 board (Tx) connected to the Zedboard FPGA board through Pmod connectors.

Figure 7a,b illustrate the FPGA’s subsystem layout and the devices used for each subsystem, respectively. The FPGA controls the DAC and four ADCs on the PL side, storing signals in FIFO memory. The PS side facilitates data transfer from the PL to the host PC by moving data from the PL to Random-Access Memory (RAM) via Direct Memory Access (DMA), then sending it to the host PC over Ethernet. Additionally, the PS side initiates the trigger signal for DAC transmission and stores the ADC-recorded signals, enabling the synchronized generation and recording of the Lamb wave signals.

The host computer (PC) has two main functions: signal display and data communication. MATLAB software is used for signal visualization. Communication between the FPGA and the host PC occurs via Ethernet, allowing data transfer from the RAM to the PC.

When the MATLAB software initiates, a trigger signal is sent from the host PC to the PS section of the ZedBoard FPGA through Ethernet, which then relays the signal to the PL modules. This process initiates the generation of Lamb wave signals by the DAC and the recording of signals by the ADCs, which are subsequently stored in RAM. The stored signals are transferred to the host PC via Ethernet, as depicted in Figure 7a.

Once the setup was thoroughly tested and validated, the experiments commenced. The plates were isolated from the table surface to minimize environmental interference, such as vibrations from nearby equipment. PZT sensors were positioned at each corner of the plates using ultrasonic gel to ensure maximum coverage. The gel was reapplied every session, and at each session, we recorded data up to 40 times from all sensors before the gel dried. In this process, 34 recordings were used for data processing from a total of 40 recordings. Then, there was one dataset for each session.

**Figure 8:**
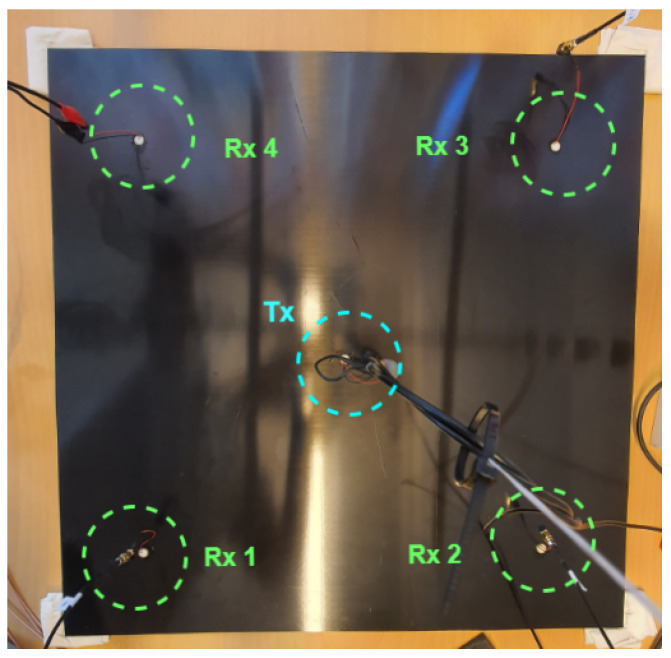
Experimental setup of a specimen with the transmitter (Tx) placed in its center and four receivers (Rx) positioned at its corners, connected to the receiver boards (Source: [33]).

The sensor layout on a specimen, with the transmitter at the center and receivers at each corner, is shown in Figure 8. A sufficient distance between each receiver and the plate edges was maintained to avoid the border effects typical with this material type [37].

**Figure 9:**
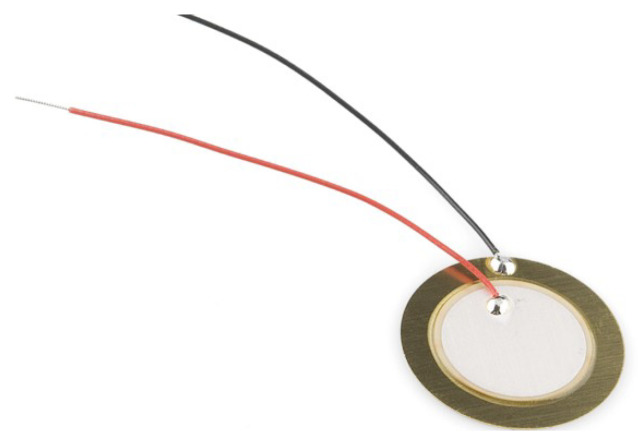
PZT sensor with 10 mm diameter patch.

The transmitter (Tx) and receivers (Rx) have piezoelectric sensor patches with a diameter of 10 mm, as depicted in Figure 9.

## 4. System’s Development: Digital Signal Processing

After developing the data acquisition system, the next step is digitally processing the receiver signals. The first step involved removing the DC offset of approximately 1.25 V, which varied slightly among receivers. Following this, the Discrete Wavelet Transform (DWT) method was applied for signal filtering, as DWT is a recognized standard for filtering Lamb wave signals [38,39].

Each receiver data record contains 8176 samples, each collected every 1 μs, resulting in a sampling frequency of 1 MHz. In DWT processing, “levels” refer to stages where the signal is repeatedly decomposed into low-frequency (approximation) and high-frequency (detail) components, with each level providing a different resolution. For this signal processing task, a 3-level DWT decomposition was applied.

**Figure 10:**
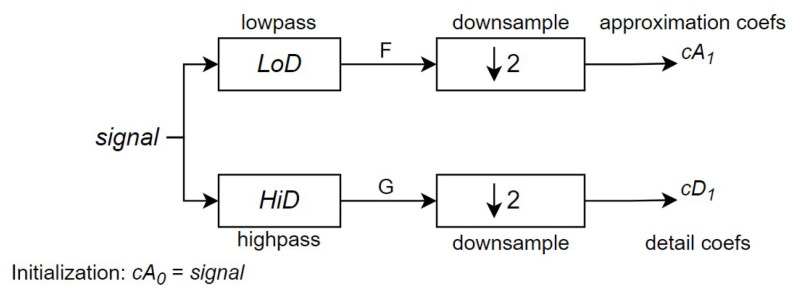
First stage of the DWT algorithm (Source: adapted from [41]).

For a signal of length *N*, the DWT can decompose up to $log_{2}N$ stages. Starting with signal, the first stage generates two sets of coefficients: approximation coefficients $cA_{1}$ and detail coefficients $cD_{1}$ (refer to Figure 10). This is achieved by convolving the signal with a low-pass filter LoD and a high-pass filter HiD, followed by dyadic decimation (downsampling by a factor of two) to obtain the approximation and detail coefficients, respectively [40]. Figure 10 illustrates the first stage of this process, where *F* is the output of the convolution between the input signal and the low-pass filter LoD, and *G* represents the output from the convolution with the high-pass filter HiD. With filters of length 2n, if the signal has *N* samples, the lengths of *F* and *G* are N+2n−1, and the lengths of $cA_{1}$ and $cD_{1}$ are $\frac{N-1}{2}+n$.

**Figure 11:**
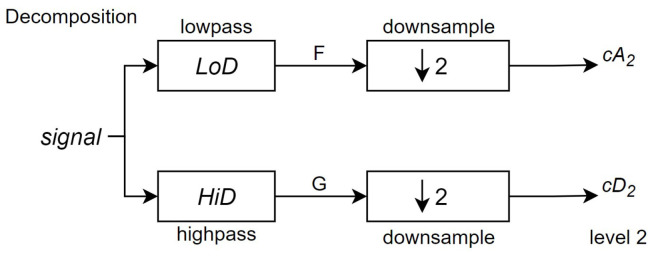
Second stage of the DWT algorithm, which is also the first stage of its decomposition process (Source: adapted from [41]).

**Figure 12:**
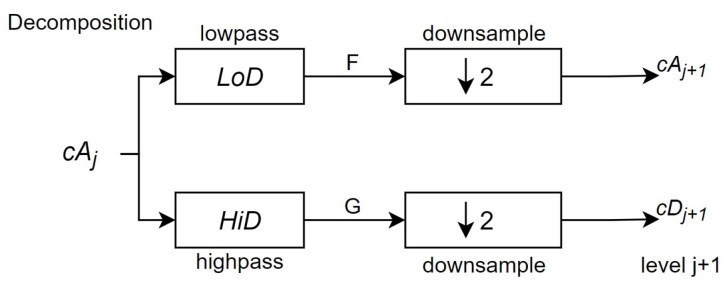
Last stage of the decomposition process for a one-dimensional DWT (Source: adapted from [41]).

The next stage splits the approximation coefficients $cA_{1}$ in to two parts using the same scheme, replacing signal by $cA_{2}$, producing $cA_{2}$ and $cD_{2}$, as shown in Figure 11, until it reaches the desired level *j*, as shown in Figure 12—which in this system is three.

**Figure 13:**
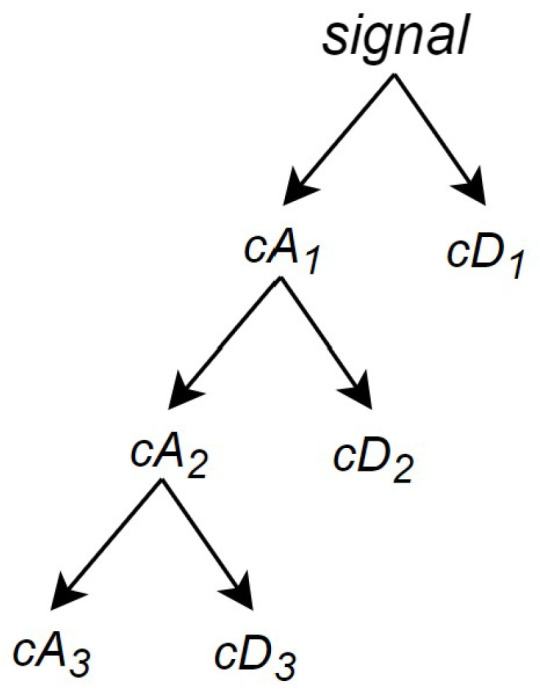
DWT coefficient decomposition structure (Source: adapted from [41]).

Then, the wavelet decomposition of signal analyzed at level *j* has the structure $\left[cA_{j},cD_{j},...,cA_{1},cD_{1}\right]$, and this structure is illustrated in Figure 13, where j=3.

The Daubechies wavelet type was selected due to its orthogonality, which enables perfect signal reconstruction without information loss. The smooth characteristics of Daubechies wavelets make them well suited for applications like image compression, denoising, and other tasks where maintaining signal or image smoothness is essential. Additionally, Daubechies wavelets are available in various orders (dbN, where *N* represents the order), allowing for flexibility in selecting a wavelet that aligns best with specific application needs. For instance, db1 corresponds to the Haar wavelet (a basic step function), while higher order options like db4, db6, and beyond provide additional vanishing moments and enhanced smoothness, enabling users to tailor the wavelet to their precise signal processing requirements. In this setup, db4 was chosen as it offered optimal denoising, preserving the key signal features.

**Figure 14:**
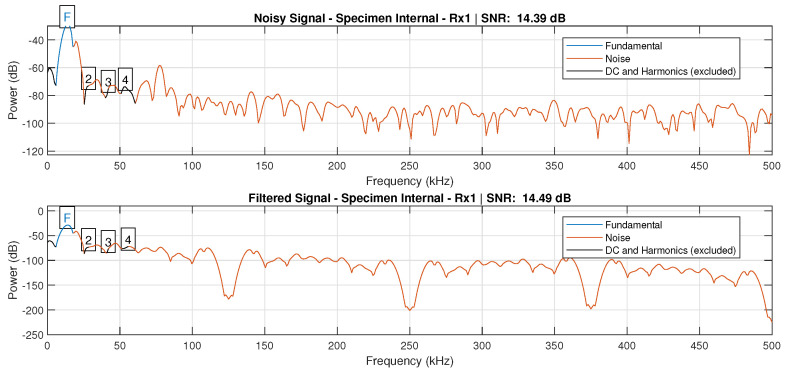
Comparison between the SNR values of the signal Rx1 with and without the 3−level db4 DWT filtering, where the signal was recorded from the plate with internal defects.

The signal-to-noise ratio (SNR) was evaluated to assess the effectiveness of this filtering setup, as depicted in Figure 14.

**Figure 15:**
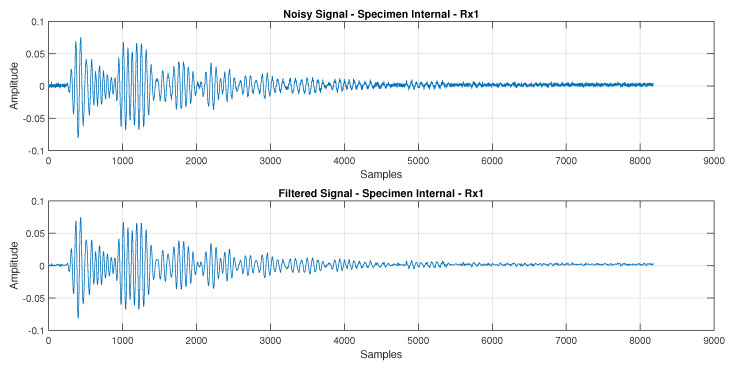
Comparison between the signal Rx1 with and without the 3−level db4 DWT filtering, where the signal was recorded from the plate with internal defects.

**Figure 16:**
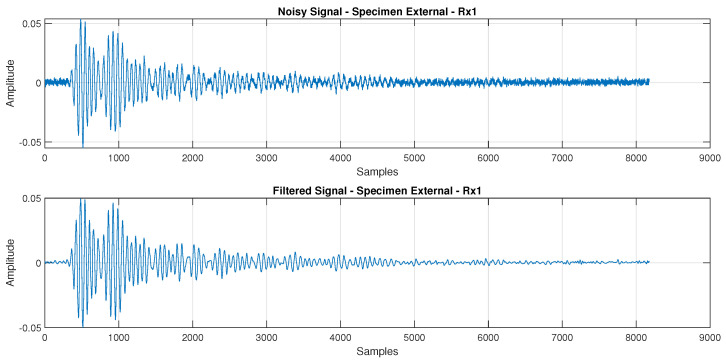
Comparison between the signal Rx1 with and without the 3−level db4 DWT filtering, where the signal was recorded from the plate with external defects.

Figure 15 and Figure 16 display the reconstructed signal following the 3-level db4 DWT decomposition. These figures illustrate the denoised Lamb wave behavior on plates with internal defects and surface defects, respectively.

After filtering the signals, feature extraction was the next digital signal processing (DSP) step. After careful analysis, the power spectral density (PSD) was chosen for extraction from the Lamb wave signals, as it clearly represented the composite material’s mechanical signature.

To summarize the PSD calculation, it is defined as the Discrete-time Fourier Transform (DTFT) of the covariance sequence r(k), as indicated in Equation (1) [42].

(1) $$\Phi \left(\omega \right)=\sum_{k=-\infty }^{\infty }r\left(k\right)e^{-i\omega k}$$

where $r\left(k\right)=E\{y\left(t\right)y^{*}\left(t-k\right)\}$ is defined as the covariance function of y(t); in this case, y(t) is the filtered signal obtained in the last step. E{·} denotes the expectation operator.

**Figure 17:**
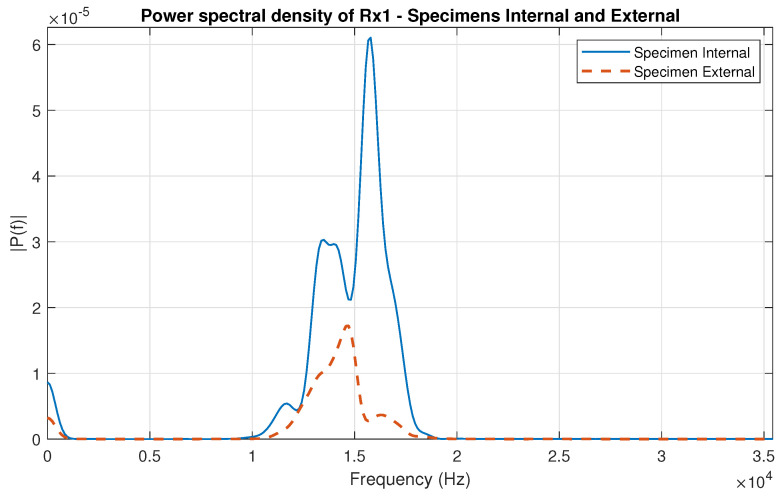
PSD of the filtered signal from receiver Rx1, where the recorded signal was obtained from the specimens with internal and external defects.

Figure 17 shows the power spectrum analysis of the filtered data from receiver Rx1 recorded from internal and external specimens.

The power spectral density matrices contain half the sample count of the initial noisy signals, which would demand substantial hardware resources for the subsequent inference phase. This made it evident that data compression was essential for the new PSD dataset, with Principal Component Analysis (PCA) selected as the compression method.

PCA is a dimensionality reduction technique that transforms large datasets into a smaller set of variables while preserving most of the original information. Although reducing the number of variables can result in minor accuracy loss, dimensionality reduction aims to achieve a balance between this loss and the benefits of a simplified dataset. Smaller datasets are more straightforward to visualize and interpret, enabling machine learning algorithms to operate more efficiently by eliminating redundant variables. PCA aims to retain as much meaningful information as possible while reducing the dataset’s dimensionality [43].

The PCA mathematical process can be summarized as solving the eigenvalue of Equation (2).

(2) Cov(X)v=λv

where *v* is an eigenvector (principal component) and λ is the corresponding eigenvalue (variance explained).

The PCA algorithm was set to retain two components, i.e., to reduce the data dimension size to two because SVM works in a two-dimensional feature space. Then, each received signal $Rx_{i}$ (i=1,2,3,4, where *i* is related to the receiver being used during the recording) is filtered and transformed into a 20×2 matrix compressed from its PSD features ($CP_{j}^{d}$), where each column *d* is related to the data matrix dimension.

$$Rx_{i}=\left[\begin{array}{cc} CP_{1}^{1} & CP_{1}^{2}\\ CP_{2}^{1} & CP_{2}^{2}\\ CP_{3}^{1} & CP_{3}^{2}\\ ... & ...\\ CP_{20}^{1} & CP_{20}^{2} \end{array}\right]$$

There were thirty-four recordings of all four receivers, which results in a 680×2 matrix for each receiver that will be separated into training and test datasets.

**Figure 18:**
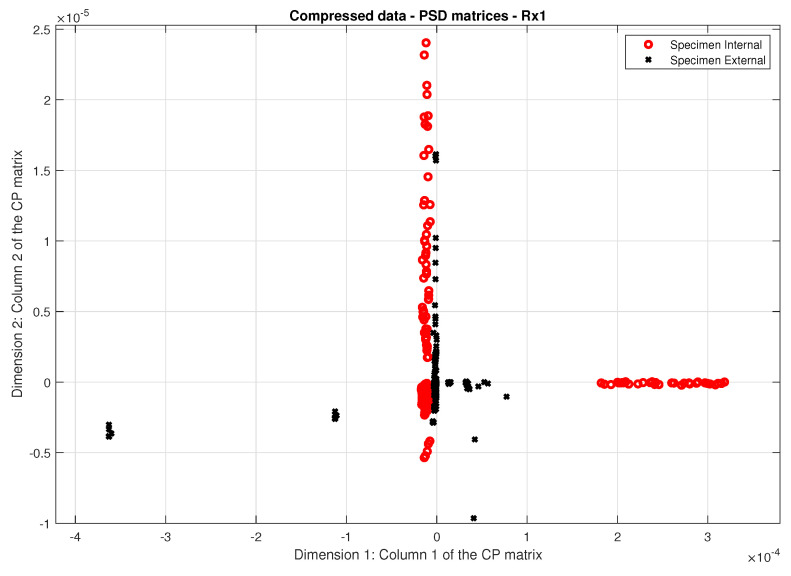
Compressed data of the PSD matrices related to the receiver Rx1 allocated in a 2−dimensional space.

Figure 18 shows the data points from the PCA application for receiver Rx1, derived from both specimens.

**Figure 19:**
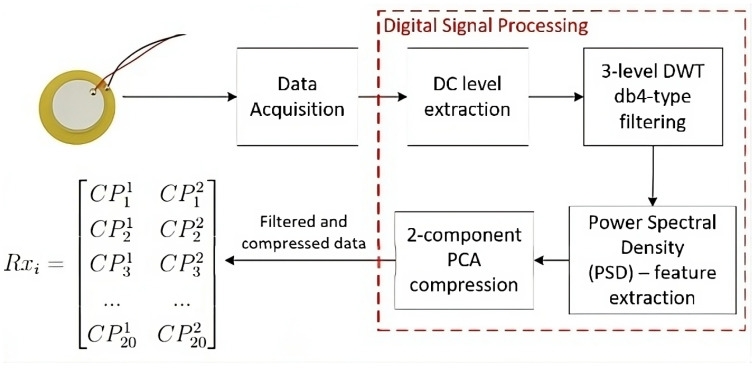
Overview of the system’s digital signal processing.

To summarize the processes involving the DSP processed in Matlab R2024a, we have Figure 19 which illustrates the step-by-step process through a data flow diagram.

1. DC level elimination of 1.25 V;
2. Filtering, using a three-level discrete wavelet transform (DWT) decomposition;
  - Setting up of the wavelets type, which was Daubechies wavelets of order 4;
  - Approximation and detail coefficients’ extraction, one for each level;
  - Soft threshold application, one threshold value for each detail coefficient;
  - Reconstruction of the signal using the filtered coefficients.
3. Parameterization: extraction of the power spectral density of the signals;
4. Data compression through the Principal Component Analysis (PCA) algorithm.

## 5. System’s Development: ML Training Phase

The dataset prepared for the machine learning implementation was split into two parts: one for training, with thirty recordings, and one for testing, with four recordings (the whole system runs each recording at once, i.e., each recording is related to the transmitter and four receivers running at once).

During the SVM training phase, the Gaussian kernel function outperformed other kernels, applied with a Kernel scale of 0.1 in Matlab R2024a using the *fitcsvm()* function. Notably, a supervised learning approach was used, where a target column vector with labels was included for each training data point in the algorithm.

Additionally, an outlier detection phase was conducted to identify outlier test data points, with outliers marked as ‘1’ and non-outliers as ‘0’.

Once the best training patterns were found and the outliers were pointed out, the training pattern data and the [1,0] outlier matrix were saved for the hardware inference phase. There are four input matrices from the training patterns: the support vectors in the matrix $SV_{N\times 2}$, the alpha values in the matrix $\alpha_{N\times 2}$, the sigma values in the matrix $Sigma_{2\times 2}$, and the Bias value, where N=135. The matrix $Outliers_{M\times 1}$ is from the outlier detection phase. Finally, the test dataset is allocated in the matrix $Test_{M\times 2}$, where M=80 (twenty compressed PSD data points from each recording times four recordings).

$$SV_{N\times 2}=\left[\begin{array}{cc} SV_{11} & SV_{12}\\ SV_{21} & SV_{22}\\ SV_{31} & SV_{32}\\ ... & ...\\ SV_{N1} & SV_{N2} \end{array}\right]\;\alpha_{N\times 1}=\left[\begin{array}{c} \alpha_{11}\\ \alpha_{21}\\ \alpha_{31}\\ \ldots \\ \alpha_{N1} \end{array}\right]\;{\mathrm{Sigma}}_{1\times 2}=\left[\begin{array}{cc} Sigma_{11} & Sigma_{12} \end{array}\right]\;\begin{array}{c} Bias_{1\times 1}=\left[Bias\right] \end{array}\;\;{\mathrm{Test}}_{M\times 2}=\left[\begin{array}{cc} Test_{11} & Test_{12}\\ Test_{21} & Test_{22}\\ Test_{31} & Test_{32}\\ \ldots & \ldots \\ Test_{M1} & Test_{M2} \end{array}\right]\;{\mathrm{Outliers}}_{M\times 1}=\left[\begin{array}{c} OutlierTest_{11}\\ OutlierTest_{21}\\ OutlierTest_{31}\\ \ldots \\ OutlierTest_{M1} \end{array}\right]$$

**Figure 20:**
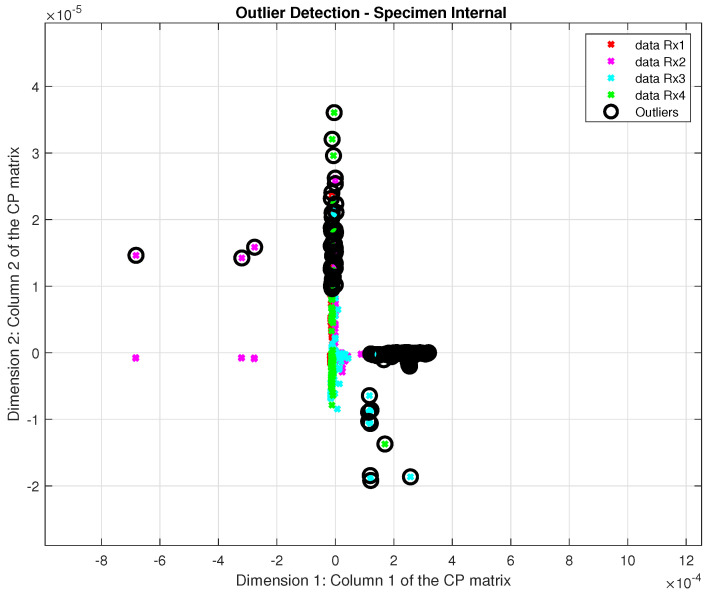
Detected outliers in the specimen internal.

**Figure 21:**
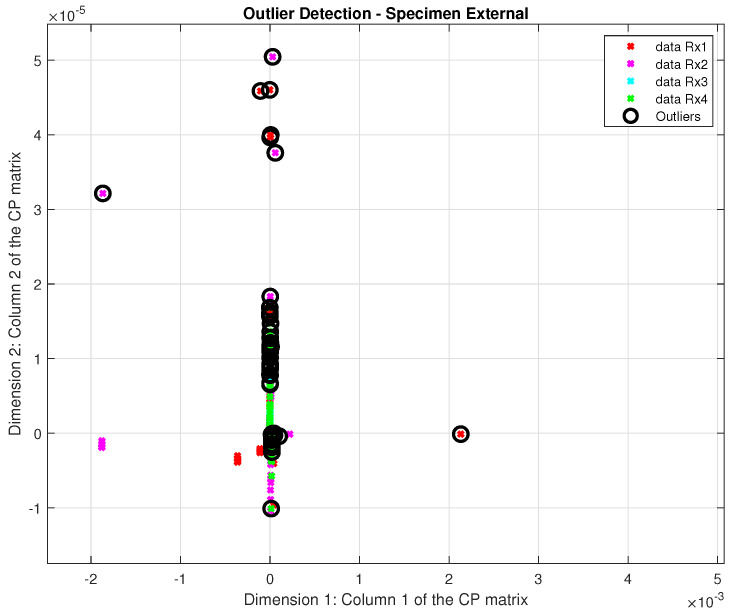
Detected outliers in the specimen external.

In the outlier detection phase, up to 8% of the dataset was identified as outliers, a relatively high percentage for this type of pattern recognition system, highlighting the need for an effective solution to address outliers. Figure 20 and Figure 21 illustrate the presence of this issue in both specimens, those with internal and external defects, respectively.

In this case, outliers can have important information about new damage or very minor damage. Then, our approach was designed not to remove outliers but to handle them effectively within the classification process. Specifically, outliers were classified using the Mahalanobis distance during the SVM classification phase, while non-outlier data were classified using the standard Euclidean distance. Furthermore, if a coefficient was identified as an outlier, its entire recording was treated as compromised; i.e., all its coefficients were classified using the Mahalanobis distance to ensure consistency and account for their interdependence.

## 6. System’s Development: Ml Inference Phase

**Figure 22:**
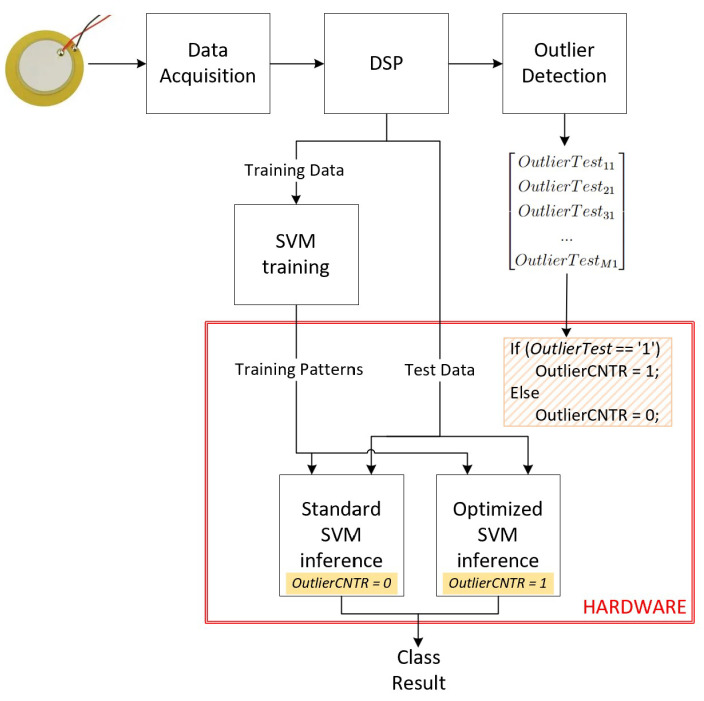
Overview of the system’s ML inference phase implemented on hardware.

The SVM inference phase was implemented on an FPGA, specifically the Zedboard ZYNQ-7 ZC702, using Verilog Hardware Description Language (HDL) and an 18-bit fixed-point format for all input data. Figure 22 presents the components of the SVM inference phase in detail with an overview of the previous stages of the system. It is possible to observe the difference between the standard method, based on Euclidean distance, and the new approach in this paper, which uses Mahalanobis distance implemented on hardware. The key distinction is having the optimized SVM inference block to solve the outliers instead of using only the standard SVM inference based on the Euclidean distance.

In standard SVMs, Euclidean distance is commonly used to construct the margin between classes. This approach can struggle with outliers, mainly when data are non-uniformly distributed or have significant correlations between features, as in this SHM case. Switching to Mahalanobis distance can address the outlier issue more effectively by re-weighting and transforming the feature space, so outliers only have a meaningful impact if they represent significant statistical deviations. This approach can lead to a more robust and generalizable margin, particularly for data with varied correlations or scales.

Euclidean Distance: Using Euclidean distance assumes that all features are treated equally, disregarding their distributions, scales, or correlations. As a result, outliers can heavily influence the decision boundary, potentially misrepresenting natural data variations or noise in high-dimensional space.

(3) $$d_{L}\left(x,y\right)=\sqrt{{\left(x-y\right)}^{T}\left(x-y\right)}$$

where *x* is the input data on *x*-axis and *y* is the same input data on *y*-axis.

Mahalanobis Distance:Mahalanobis distance takes into account the covariance structure of the data, effectively normalizing for features with varying variances or correlations. This approach “scales” the data based on their natural spread, ensuring that features with more significant variability or stronger correlations contribute less to the calculated distance.

(4) $$d_{M}\left(x,y\right)=\sqrt{{\left(x-y\right)}^{T}S^{-1}\left(x-y\right)}$$

where *x* is the input data on x-axis, *y* is the same input data on y-axis, and *S* is the covariance matrix of the input data.

In practice, estimating the covariance matrix *S* from the data is possible. This is completed during the inference phase using the sample covariance of the data, as shown in Equation (5).

(5) $$S=\frac{1}{n-1}\sum_{i=1}^{n}\left(x_{i}-\mu \right){\left(x_{i}-\mu \right)}^{T}$$

where $x_{i}$ are the test samples and μ is the mean of the samples.

**Figure 23:**
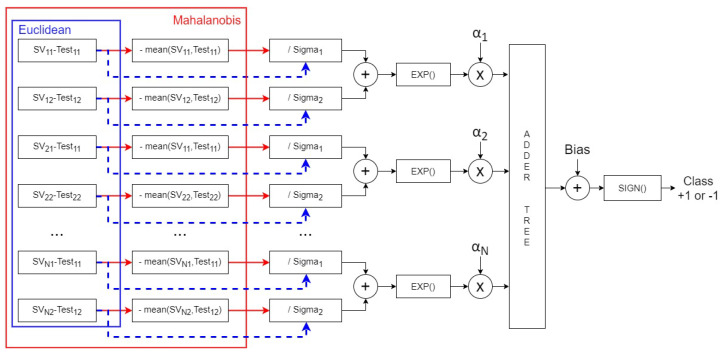
Block diagram of the HDL components for the SVM datapath implementation.

For this reason, we have a new block included in the SVM inference datapath: the block $mean(SV_{N\times 2}-Test_{M\times 2})$, as shown in Figure 23.

Algorithm 1 outlines the step-by-step datapath for the SVM inference phase using Mahalanobis distance calculation. In this process, the “MEAN” block is activated when the Outliers matrix indicates a logic level of “1”, signifying that the respective Test data point is an outlier. This phase operates through a pipelined datapath managed by a Finite State Machine (FSM).
**Algorithm 1** SVM inference phase based on Mahalanobis.**Require:** SVs;Alpha;Bias;Sigma;Test;Mean1:**for** cont = 1:size(Test,1) **do**2:    **for** i = 1:size(SVs,1) **do**3:        $aux=({\left(SVs\left(i,1\right)-Test\left(cont,1\right)-Mean\left(i,1\right)\right)}^{2})/Sigma\left(1\right)$4:        $aux1=({\left(SVs\left(i,2\right)-Test\left(cont,2\right)-Mean\left(i,2\right)\right)}^{2})/Sigma\left(2\right)$5:        SqDif(i)=sqrt(aux+aux1)6:        EXPin(i)=−SqDif(i)7:        EXPout(i)=exp(EXPin(i))8:        AlphaMult(i)=Alpha(i)∗EXPout(i)9:    **end for**10:    adderTree(cont,1)=sum(AlphaMult)11:    BiasSum(cont,1)=adderTree(cont,1)+Bias12:**end for**13:**for** i = 1:size(BiasSum,1) **do**14:    **if** BiasSum(i,1) >= 0 **then**15:        Class(i,1)=116:    **else**17:        Class(i,1)=018:    **end if**19:**end for**

**Figure 24:**
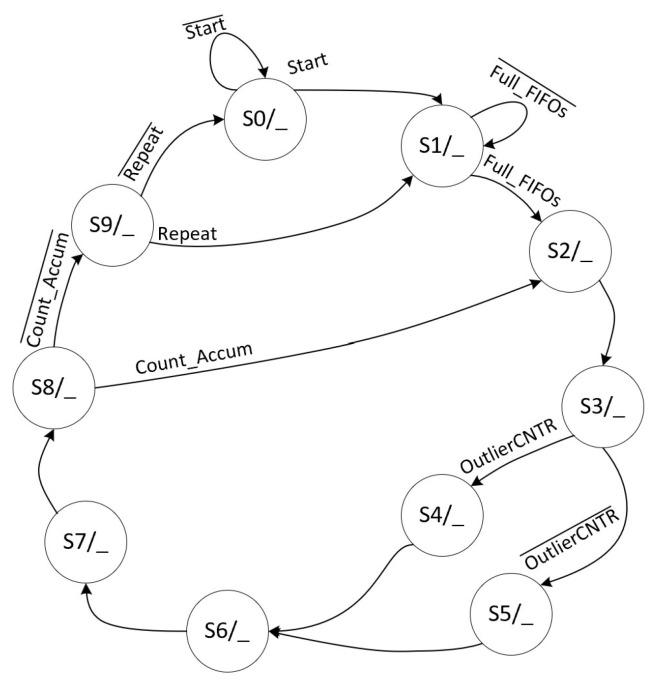
FSM specification responsible for controlling the optimized SVM inference datapath using a method based on the Mahalanobis distance calculation.

**Figure 25:**
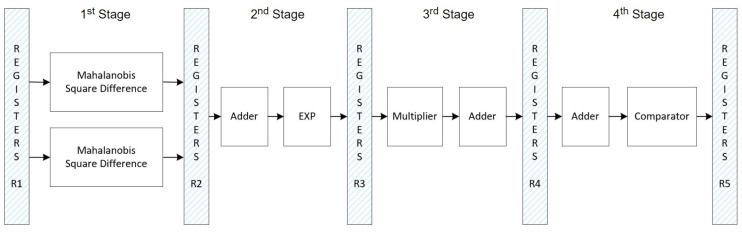
Optimized SVM inference datapath controlled by the FSM of Figure 24 based on the Mahalanobis distance calculation.

The FSM that controls these functions in Algorithm 1 has ten states, as shown in Figure 24, and its datapath is shown in Figure 25, where each FSM stage is responsible for controlling parts of the SVM datapath. Each stage has the following functions:

- S0: Initializes the entire circuit;
- S1: Loads the FIFOs;
- S2: Calculates the EuclideanSquareDifference;
- S3: Checks the Outliers matrix; when the data are “1”, then the FSM goes to stage 4; otherwise, it goes to stage 5;
- S4: Calculates the MahalanobisSquareDifference equation;
- S5: Calculates the EuclideanSquareDifference equation;
- S6: Calculates the adder and EXP blocks;
- S7: Calculates the multiplication by Alpha;
- S8: Calculates the AdderTree block of the datapath, which is the 3rd stage of the pipelined datapath;
- S9: Calculates the adder and SGN blocks of the datapath.

The signal Full_FIFOs checks if both FIFOs are full. The signal

Count_Accum checks if the accumulator block is complete, i.e., if it has been processed 135 times because it depends on the process of each machine.

There are two *Mahalanobis Square Difference* blocks because there are two dimensions in the SVM feature space (the high-dimension space that SVM uses to separate two classes through the optimal hyperplane [44]), and each block processes the support vector values for its respective dimension.

### Comparison: Standard SVM vs. Optimized SVM

**Figure 26:**
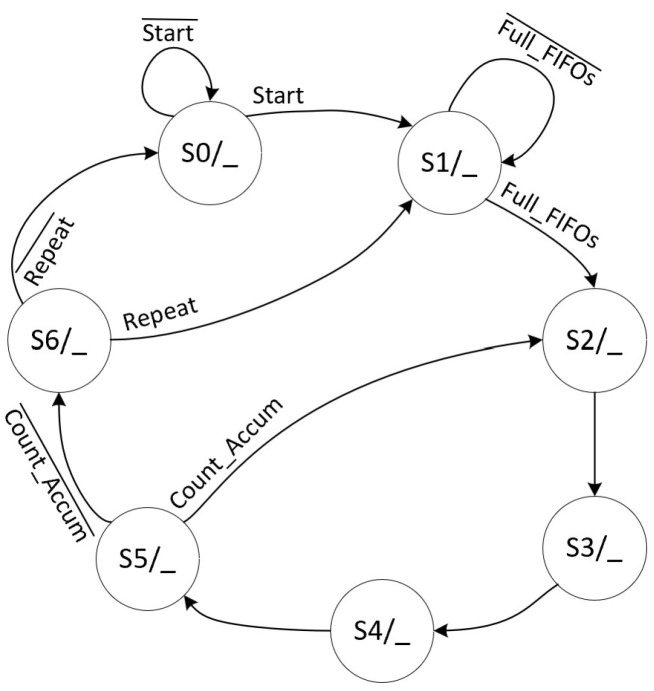
FSM specification responsible for controlling the SVM inference datapath using the standard Euclidean method.

**Figure 27:**
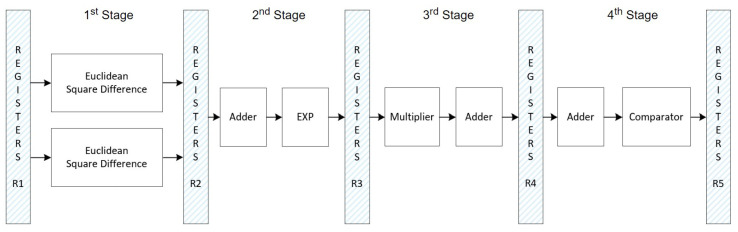
SVM inference datapath controlled by the FSM of Figure 26, using the standard Euclidean method.

A second implementation was created using the standard Euclidean distance in the SVM algorithm for comparison with the optimized version that incorporates Mahalanobis distance. The FSM consists of seven states in this standard SVM setup, illustrated in Figure 26. The associated datapath is shown in Figure 27, where each FSM stage manages specific parts of the SVM datapath. The functions of each stage are as follows:

- S0: Initializes the entire circuit;
- S1: Loads the FIFOs;
- S2: Calculates the EuclideanSquareDifference;
- S3: Calculates the adder and EXP blocks;
- S4: Calculates the multiplication by Alpha;
- S5: Calculates the AdderTree block of the datapath, which is the third stage of the pipelined datapath;
- S6: Calculates the adder and SGN blocks of the datapath.

The signal Full_FIFOs checks if both FIFOs are full. The signal Count_Accum checks if the accumulator block is complete, i.e., if it has been processed 135 times because it depends on the process of each machine. The datapath of Figure 27 has the same structure as in Figure 25, where the first stage is different.

## 7. Results and Discussions

As expected, the Mahalanobis distance-based solution demonstrated improved success rates, as detailed in Table 1. This table compares success rates from the training phase conducted in Matlab with those from the FPGA inference phases across the two specified classes.

- **Class 1:** Defects located on the internal part of the specimen;
- **Class 2:** Defects located on the external side of the specimen (on its surface).

The hardware architectures implemented follow two patterns described in Section 6: the standard Euclidean distance method and the optimized Mahalanobis distance solution. The table below illustrates that the optimized solution achieves a higher success rate than the standard implementation.

### 7.1. Comparison: Standard SVM vs. Optimized SVM

Table 2 compares the results of the standard and optimized architectures. As anticipated, the optimized version shows increased area, latency, and power consumption due to the addition of the mean subtraction block, as shown in Figure 23.

### 7.2. Comparison: Proposed System vs. Other Researchers Proposal

Mardanshahi et al. [45] focused on using guided wave propagation combined with machine learning techniques, specifically SVM and neural networks, to detect and classify the structure matrix cracking in glass/epoxy cross-ply laminated composites. The researchers fabricated samples with varying densities of matrix cracks, using linear discriminant analysis to reduce data dimensionality before classification. Their results showed that SVM achieved a high accuracy rate of 91.7%, outperforming other study models.

The proposed work uses Lamb waves for real-time SHM in composites through FPGA hardware, emphasizing Mahalanobis distance in SVM to manage outliers. In contrast, Mardanshahi et al. [45] also use wave propagation but focus on matrix cracking and apply dimensionality reduction before SVM classification. Mardanshahi et al. achieved a detection accuracy of 91.7% using SVM, slightly lower than the reported 96.25% for internal defects and 97.5% for external defects. The proposed study in this paper is implemented on FPGA hardware with enhanced SVM outlier handling, which makes it suitable for dynamic, real-time SHM applications. It addresses practical limitations that Mardanshahi’s approach may face in real-world SHM contexts.

## 8. Conclusions

The system is designed to locate damage within the composite structure by processing data sequentially from each receiver (Rx1 to Rx4). This “receiver-by-receiver” monitoring approach allows the system to associate damage detection results directly with the position of the active receiver during the machine learning (ML) inference phase. By having one receiver positioned in each quadrant of the panel, the system achieves a basic level of spatial localization, enabling it to identify the damaged region based on the receiver’s location. When the ML classifier outputs a positive result indicating damage, the system correlates this output with the specific receiver channel to determine the approximate location of the defect. While the current implementation provides coarse localization within the receiver quadrants, the precise measurement of damage size and more granular localization remain areas for future development. This will involve refining the sensor network, incorporating additional receivers, and leveraging advanced signal processing techniques to enhance spatial resolution and damage characterization.

This study developed an FPGA-based SHM system using an optimized SVM algorithm for the real-time detection of internal and external defects in composite materials. The system achieved high classification accuracy by integrating an operation based on the Mahalanobis distance into the SVM inference phase.

Unlike the standard Euclidean distance, Mahalanobis distance considers feature correlations and variability in data distributions, allowing for more precise and resilient outlier detection. This makes it particularly effective for machine learning implementations on hardware, especially in SHM systems, where accurate defect detection is sensitive to outliers. Mahalanobis distance refines the decision boundary based on the data’s covariance structure, reducing the likelihood of misclassifying natural data variations as outliers, a limitation seen with Euclidean distance.

Compared with other outlier detection approaches, the FPGA-based hardware implementation of Mahalanobis distance stands out in efficiency and accuracy. The optimized solution reached success rates of 96.25% for internal defect detection and 97.5% for external defects, surpassing the standard Euclidean approach, which achieved 90% and 86.25%, respectively. While the optimized method introduced minor increases in power consumption and latency due to the additional computations for Mahalanobis distance, this trade-off was well justified by the marked improvement in detection performance.

The FPGA implementation enabled fast, low-power processing suitable for real-time monitoring. This approach reduces latency, making it practical for SHM applications where timely data processing is essential, particularly in aerospace. The results confirm that Mahalanobis-enhanced SVM improves defect detection in composite materials, offering a robust and efficient solution for SHM in critical structural applications.

Future work is intended to advance the front-end system: data acquisition. An ASIC version can be developed to miniaturize this part of the system. In addition, the FPGA’s reconfigurability feature can be used to improve damage characterization, offering more details about the damages.

While this study focuses on a single transmitter and four receivers to validate the concept, future work will explore scalability by integrating multiple transmitters and expanding the receiver network to evaluate system performance on larger, more complex structures. Additional studies will also investigate the impact of sensor placement on signal quality, coverage efficiency, and robustness against noise and edge effects.

## Figures and Tables

**Table 1:** Table of comparison between results from software and hardware implementations (using the standard and optimized architectures).

Software: Matlab R2024a		
Training phase		99.25%
Inference phase	Class 1	98.75%
	Class 2	98.125%
Hardware		
Standard inference phase—Euclidean	Class 1	90%
	Class 2	86.25%
Optimized inference phase—Mahalanobis	Class 1	96.25%
	Class 2	97.5%

**Table 2:** Table of comparison between results from the standard and optimized implementations.

Features	Architectures	
	Standard	Optimized
Throughput	64 ns	68 ns
Latency	28.48 μs	30.26 μs
LUTs	968/53,200	1051/53,200
Flip-flops	551/106,400	574/106,400
DSP	3/220	5/220
Power	5 mW	6 mW

## Data Availability

Data are contained within the article.
